# Genome-wide association analysis of heifer livability and early first calving in Holstein cattle

**DOI:** 10.1186/s12864-023-09736-0

**Published:** 2023-10-21

**Authors:** Yahui Gao, Alexis Marceau, Victoria Iqbal, Jose Antonio Torres-Vázquez, Mahesh Neupane, Jicai Jiang, George E. Liu, Li Ma

**Affiliations:** 1 Department of Animal and Avian Sciences, University of Maryland, Room 2123, 8127 Regents Drive, College Park, MD 20742 USA; 2 Animal Genomics and Improvement Laboratory, BARC, USDA-ARS, Beltsville, MD 20705 USA; 3 Department of Animal Science, North Carolina State University, Raleigh, 27695 USA

**Keywords:** Dairy Cattle, Heifer, Fertility, Disease, GWAS, TWAS

## Abstract

**Background:**

The survival and fertility of heifers are critical factors for the success of dairy farms. The mortality of heifers poses a significant challenge to the management and profitability of the dairy industry. In dairy farming, achieving early first calving of heifers is also essential for optimal productivity and sustainability. Recently, Council on Dairy Cattle Breeding (CDCB) and USDA have developed new evaluations of heifer health and fertility traits. However, the genetic basis of these traits has yet to be thoroughly studied.

**Results:**

Leveraging the extensive U.S dairy genomic database maintained at CDCB, we conducted large-scale GWAS analyses of two heifer traits, livability and early first calving. Despite the large sample size, we found no major QTL for heifer livability. However, we identified a major QTL in the bovine MHC region associated with early first calving. Our GO analysis based on nearby genes detected 91 significant GO terms with a large proportion related to the immune system. This QTL in the MHC region was also confirmed in the analysis of 27 K bull with imputed sequence variants. Since these traits have few major QTL, we evaluated the genome-wide distribution of GWAS signals across different functional genomics categories. For heifer livability, we observed significant enrichment in promotor and enhancer-related regions. For early calving, we found more associations in active TSS, active Elements, and Insulator. We also identified significant enrichment of CDS and conserved variants in the GWAS results of both traits. By linking GWAS results and transcriptome data from the CattleGTEx project via TWAS, we detected four and 23 significant gene-trait association pairs for heifer livability and early calving, respectively. Interestingly, we discovered six genes for early calving in the Bovine MHC region, including two genes in lymph node tissue and one gene each in blood, adipose, hypothalamus, and leukocyte.

**Conclusion:**

Our large-scale GWAS analyses of two heifer traits identified a major QTL in the bovine MHC region for early first calving. Additional functional enrichment and TWAS analyses confirmed the MHC QTL with relevant biological evidence. Our results revealed the complex genetic basis of heifer health and fertility traits and indicated a potential connection between the immune system and reproduction in cattle.

**Supplementary Information:**

The online version contains supplementary material available at 10.1186/s12864-023-09736-0.

## Background

Heifers are young female cows that have not yet given birth to a calf and are the future of the dairy herd. Heifer health and fertility are crucial for the success of a dairy farm, as it directly affects milk production and the sustainability of a farm [1]. Healthy heifers are more likely to produce more milk once they begin lactating. In addition, heifers need to be healthy and well-cared for to conceive and carry a calf to term successfully. Unhealthy heifers may have difficulty getting pregnant, leading to lower milk production and fewer replacement cows for the farmer [2]. Diseased heifers may also require costly veterinary treatments that can add up quickly and cut into profits. Early first calving in heifers is also important for dairy farming, particularly for economic and environmental considerations, because it can reduce unproductive periods and increase lifetime production, faster generation turnover and selection progress, and improve reproductive efficiency [3]. In summary, heifer health and fertility are critical for a profitable and sustainable dairy operation [1].

The health and fertility of cows are complex issues that involve various factors, including nutrition, environment, physiology, and genetics [4–6]. Compared to cows, heifers generally encounter more challenges as heifers have not yet reached sexual maturity and are not yet capable of smooth reproduction and production. Moreover, heifers typically have additional nutritional requirements than cows, as they are still growing and developing. Despite the complexity of cattle health and fertility, many GWAS studies have been conducted to identify genomic regions and genes associated with health and fertility-related traits in cattle [7–12]. For instance, the bovine MHC region has been associated with cow livability and immune system-related diseases [7]. The *ABCC9* and *GC* genes have been associated with pregnancy rate [8], while *ARRDC3* was associated with growth and calving traits [4]. Heifer fertility and health traits are less studied than cows, mainly due to limited data availability.

Although the heritability of fertility and health traits tends to be relatively low, CDCB and the USDA Animal Genomics and Improvement Lab have been evaluating fertility and health-related traits using the large volume of data collected from the dairy industry (https://uscdcb.com/). Recently, they added heifer livability and early first calving to the evaluation system [13, 14]. Heifer livability represents the expected survival percentage of an animal’s female offspring from 2 days after birth up to 18 months of age in a herd with average management conditions. Larger, positive values of heifer livability are more favorable. It measures a heifer’s overall resistance to causes leading to mortality. Since the most common reasons for heifer death are digestive and respiratory diseases [15], heifer livability is primarily related to the resistance to these diseases and other causes of death. The heritability of heifer mortality has been estimated to be less than 1% in many studies [13, 16, 17]. Early first calving (EFC) is defined as the age at first calving. As a heifer fertility trait, the heritability of EFC is only 2–3% [14, 18, 19]. As part of the genetic evaluation process, traits have been corrected for management effect by CDCB, resulting in a PTA (Predicted Transmission Ability) that can be used directly for genetic studies. In this research, we aim to identify genes and genomic regions associated with these heifer traits using the large amount of genotype and phenotype data from the US dairy genomic database. To further boost power, we also included transcriptome and other functional genomics data for fine-mapping and validation.

## Results

### Large-sample GWAS of heifer livability and early calving

**Fig. 1:**
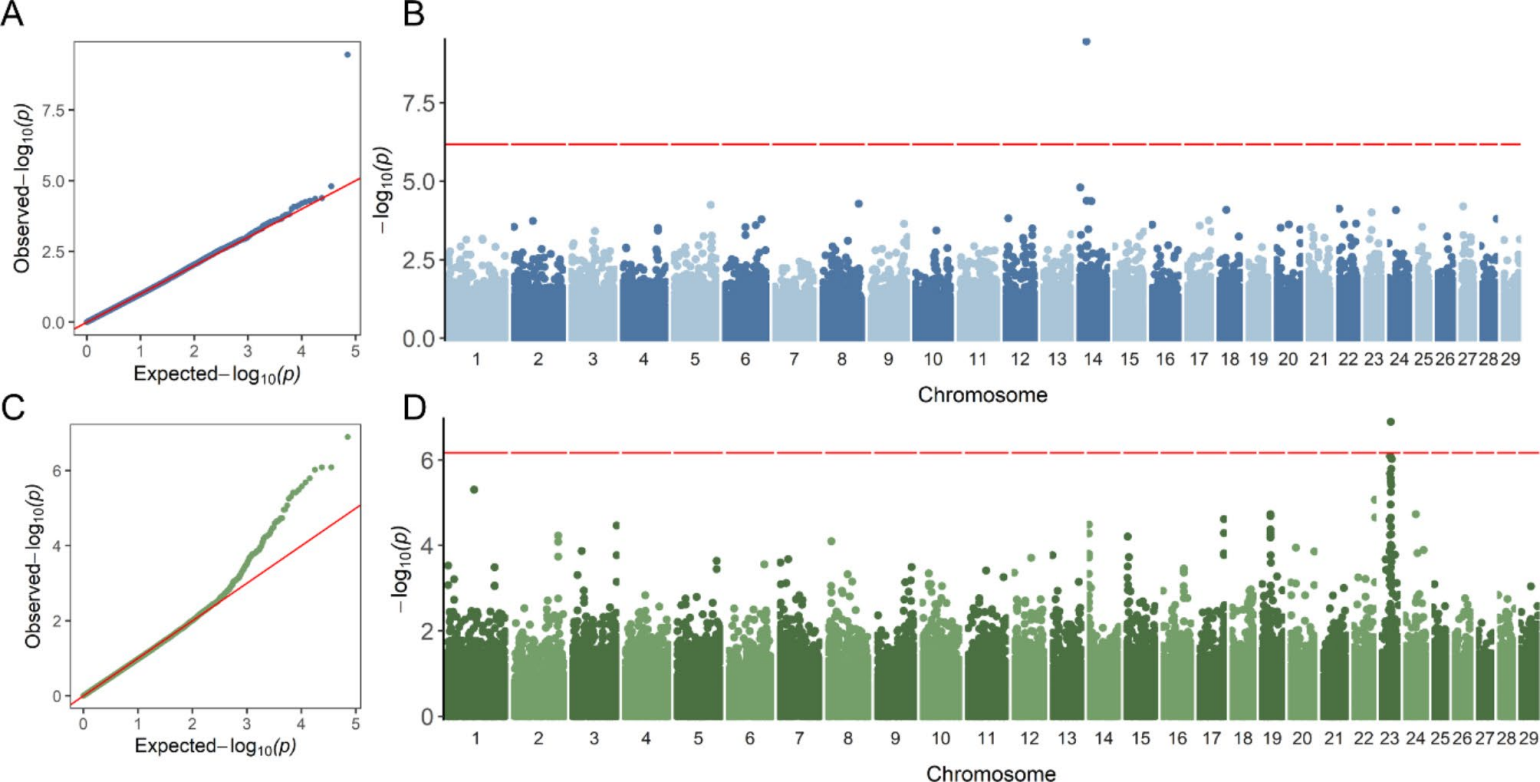
**Large-scale GWAS results of heifer livability and early calving based on the 79 K SNPs.** (**A**) Quantile–quantile (QQ) plot for heifer livability. (**B**) Manhattan plot for heifer livability. (**C**) QQ plot for early calving. (**D**) Manhattan plot for early calving. The red horizontal lines correspond to the genome-wide significance threshold

Our large-sample GWAS started with a discovery population of 3,649,734 genotyped Holstein cattle (336,386 bulls and 3,313,348 cows). After calculating deregressed predicted transmitting ability (PTA) as phenotype and editing, we included 510,318 and 768,645 animals for the GWAS of heifer livability and early first calving, respectively. All of the animals were imputed to 79 K SNPs, and we retained 73,554 SNPs after QC editing. We applied SLEMM [20] to perform the GWAS analyses that can efficiently run large-scale mixed models and incorporate variational residual variances for differential reliabilities of degressed PTAs. As a result, we found only one QTL region for each of the two traits (Fig. 1A and B C, and 1D). Nonetheless, for both traits, the *P* values for the majority of SNPs showed no inflation of test statistics and good quality of the results (Fig. 1A C). After removing SNPs with low minor allele frequency (MAF), only one SNP (ARS-BFGL-NGS-105563, *P* = 1.28e-07) passed the Bonferroni-corrected threshold for early calving (Table 1). Interestingly, this SNP is located near the Bovine MHC region on BTA 23 that encodes many fundamental molecules for regulating the immune response [21].

**Table 1:** GWAS results for two heifer traits based on 79 K SNPs

Trait	Chr	SNP	Position	*P*	MAF
Heifer Livability	5	BovineHD0500029553	102,805,184	5.66E-05	0.0988
	8	BovineHD0800030830	101,970,883	5.16E-05	0.4321
	14	Hapmap25183-BTC-049425	5,880,036	1.59E-05	0.4835
	14	BovineHD1400007185	23,060,870	3.53E-10	0.0011
	14	BovineHD1400007271	23,389,588	4.16E-05	0.0011
	14	BTA-107899-no-rs	36,267,667	4.32E-05	0.0020
	18	BovineHD1800007256	23,555,637	8.14E-05	0.3387
	22	ARS-BFGL-NGS-100995	5,061,438	7.42E-05	0.2269
	23	BovineHD2300005239	20,146,079	9.79E-05	0.4162
	24	BTA-57516-no-rs	20,681,407	8.31E-05	0.3521
	27	BovineHD2700002926	10,675,111	6.26E-05	0.3911
Early Calving	1	Hapmap38109-BTA-36588	73,971,461	4.95E-06	0.1178
	22	ARS-BFGL-NGS-67185	60,478,622	8.48E-06	0.0260
	23	BovineHD2300007231	26,926,436	8.13E-07	0.1781
	23	BTA-27247-no-rs	26,934,192	2.07E-06	0.4998
	23	BovineHD2300007469	27,446,664	8.13E-07	0.2150
	23	BovineHD2300007953	28,526,405	2.64E-06	0.3401
	23	BovineHD2300008056	28,785,343	5.63E-06	0.4924
	23	BovineHD2300008081	28,825,626	3.31E-06	0.1532
	23	ARS-BFGL-NGS-105563	29,018,391	1.28E-07	0.4395
	23	BovineHD2300008507	29,958,908	1.61E-06	0.4158
	23	ARS-BFGL-NGS-104394	30,013,004	3.85E-06	0.4494
	23	Hapmap36280-SCAFFOLD155216_10397	30,176,828	3.85E-06	0.4495
	23	BovineHD2300008966	31,163,980	9.45E-07	0.1188

Despite the few QTL regions detected in the initial GWAS, we evaluated all SNPs passing the suggestive significance levels for functional annotation analyses. For heifer livability, we obtained 118 genes located within or overlapping the vicinity of leading SNPs (< 1 Mb) using BioMart in the Ensembl database (Ensembl Genes 106; Table S1). Several genes close to the top SNPs exhibited biological relevance for cow livability, including *CHCHD7* and *PLAG1*, which are related to growth and development [22] and the *LYN* gene related to the regulation of innate and adaptive immune responses [23]. We also performed GO analysis by KOBAS [24] to determine the potential biological functions of these genes. As a result, 139 significant GO terms (*P* < 0.05) were found, with the top relevant terms being mineral absorption, homeostasis, metabolic process, and development (Table S2). According to existing studies and the cattle QTL database [25], in the upstream and downstream 1 Mb range of the top SNPs, many QTLs were previously associated with milk production, body type, and disease related traits in dairy cattle (Table S3).

For early first calving, we identified 596 genes within or near the associated SNPs (Table S4). Notably, the top associated SNPs were located within or near the bovine MHC region on BTA 23, indicating potential connections between the immune system and early first calving [26]. Many nearby genes were involved with immune functions and relevant biology for early calving, including *ABCF1*, *ABHD16A*, *AGER*, *BOLA-NC1*, *BTN1A1*, *LTA*, *LTB*, etc. We also performed the GO analysis based on these genes and detected 91 significant GO terms (*P* < 0.05) with a large proportion associated with immune processes (Table S5). Finally, previously reported QTL within 1 Mb of associated SNPs were associated with milk production, reproduction, body type, and disease-related traits in cattle (Table S6).

### Sequence-level GWAS and fine mapping of heifer livability and early calving in 27,235 bulls

**Fig. 2:**
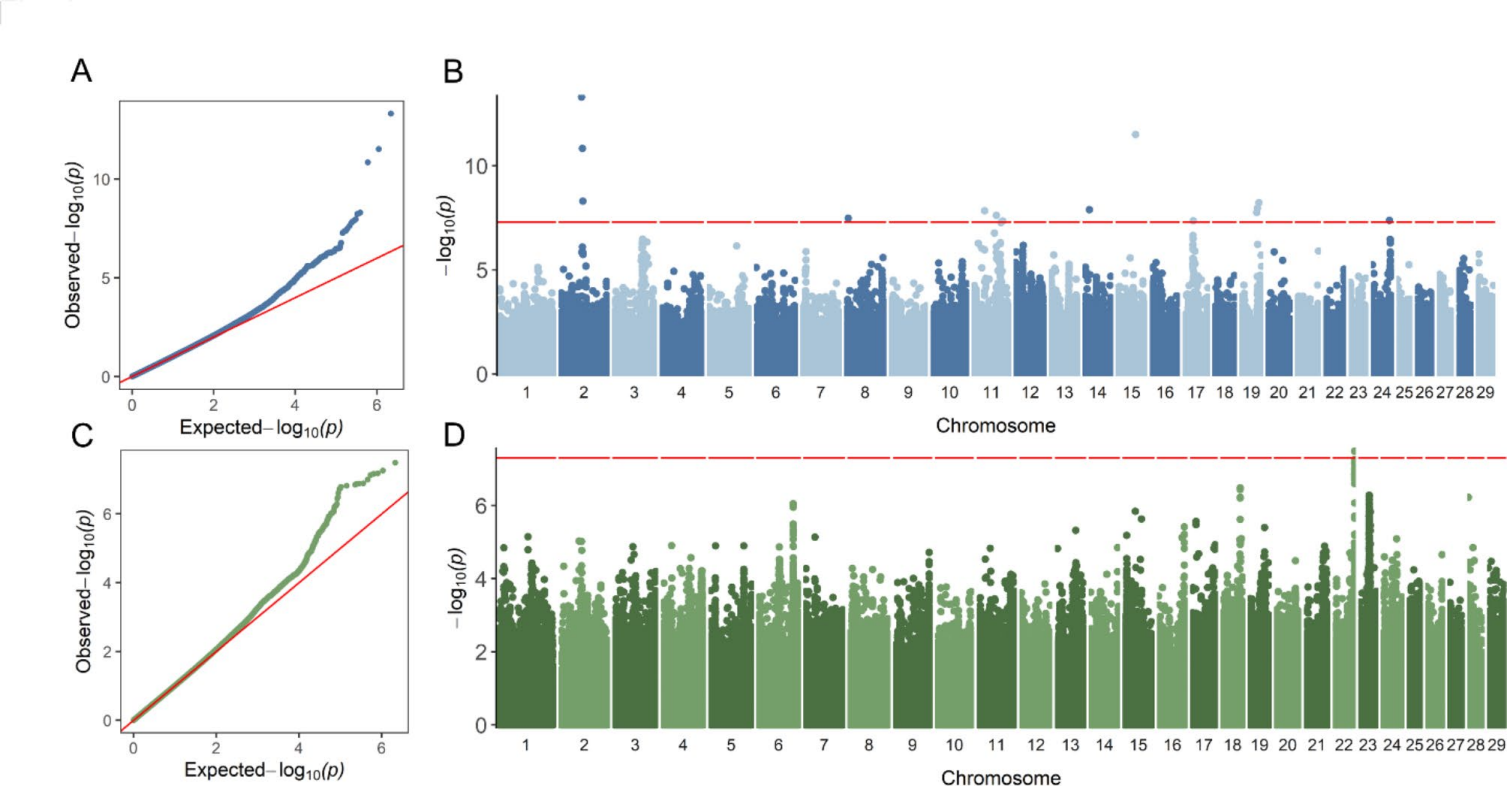
**GWAS results of heifer livability (n = 11,562) and early calving (n = 10,700) based on bulls with imputed sequence variants.** (**A**) Quantile–quantile (QQ) plot for heifer livability. (**B**) Manhattan plot for heifer livability. (**C**) QQ plot for early calving. (**D**) Manhattan plot for early calving. The red horizontal lines correspond to the genome-wide significance threshold

To refine the GWAS results, we conducted additional GWAS analyses with imputed sequence data for heifer livability and early first calving in 27,235 bulls that have highly accurate phenotypes. We used 3,148,506 imputed sequence SNPs as genotype and de-regressed PTAs as phenotype. After editing and filtering on reliability, we included 11,562 and 10,700 bulls for heifer livability and early calving, respectively. The QTL regions discovered in the large-sample GWAS were validated for both traits at the nominal significance level (Fig. 2A and B C, and 2D). Interestingly, sequence-level GWAS found some additional associations compared to low-density SNP data (Fig. 2B and D). As shown in Tables 2 and 16 SNPs passed the genome-wide threshold for heifer livability, and two SNPs passed the threshold for early calving. By checking the 1-Mb regions surrounding these associated SNPs, we identified many genes that were also detected in the large-sample GWAS, namely *MOG, OR12D2E, OR12D3, OR2H1, OR5V1, OR5V1C, OR5V2, TRIM10, TRIM15* (Table S7).

**Table 2:** GWAS results based on imputed sequence variants for heifer livability (n = 11,562) and early calving (n = 10,700)

Trait	Chr	Position	*P*
Heifer Livability	2	60,792,638	4.90E-14
	2	63,123,798	1.44E-11
	2	63,150,519	1.44E-11
	2	64,387,597	5.00E-09
	8	6,002,999	3.24E-08
	11	33,641,469	1.43E-08
	11	67,900,672	2.39E-08
	11	67,903,861	2.39E-08
	11	87,363,160	4.45E-08
	14	16,826,158	1.27E-08
	15	54,473,598	3.05E-12
	17	27,784,375	4.32E-08
	19	48,236,260	1.73E-08
	19	50,258,942	1.07E-08
	19	54,797,534	5.91E-09
	24	52,033,790	4.15E-08
Early Calving	22	60,394,806	3.26E-08
	22	60,422,561	3.26E-08

### Functional enrichment analysis

**Fig. 3:**
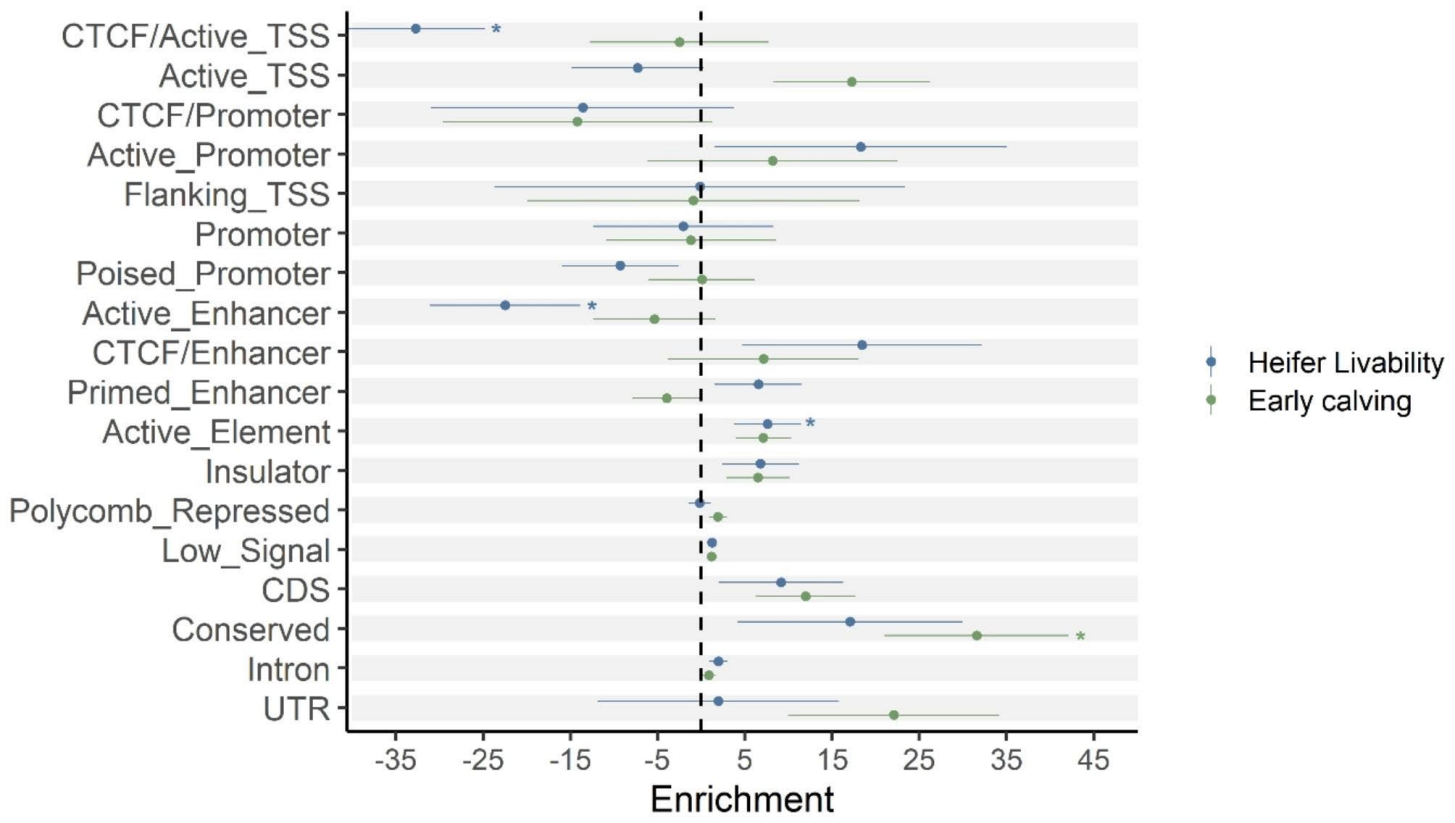
Enrichment of fine-mapping variants across functional annotations

We analyzed the enrichment of GWAS signals across SNPs in different functional genomic regions based on the 27 K bulls and imputed sequence data. We first categorized sequence variants into 14 groups based on the locations of 14 chromatin states reported previously [27], i.e., CTCF/Active_TSS, Active_TSS, CTCF/Promoter, Active_Promoter, Flanking_TSS, Promoter, Poised_Promoter, Active_Enhancer, CTCF/Enhancer, Primed_Enhancer, Active_Element, Insulator, Polycomb_Repressed, and Low_Signal. For heifer livability, we observed significant enrichment of variants in Active_Promoter, Promoter, CTCF/Enhancer, Primed_Enhancer, and Active_Element (Fig. 3). For early calving, we observed significant enrichment of associated variants in Active_TSS, Active_Element, and Insulator (Fig. 3).

We further investigated the enrichment of variants concerning their genomic locations (conserved) and genic annotations (CDS, intron, and UTR) inferred by SnpEff [28]. As a result, we observed significant enrichment of CDS and conserved variants in the GWAS results of both traits. For heifer livability, we observed enrichment of intron variants (Fig. 3). And for early calving, we observed significant enrichment of variants in the UTR regions (Fig. 3).

### Transcriptome-wide association study (TWAS)

**Fig. 4:**
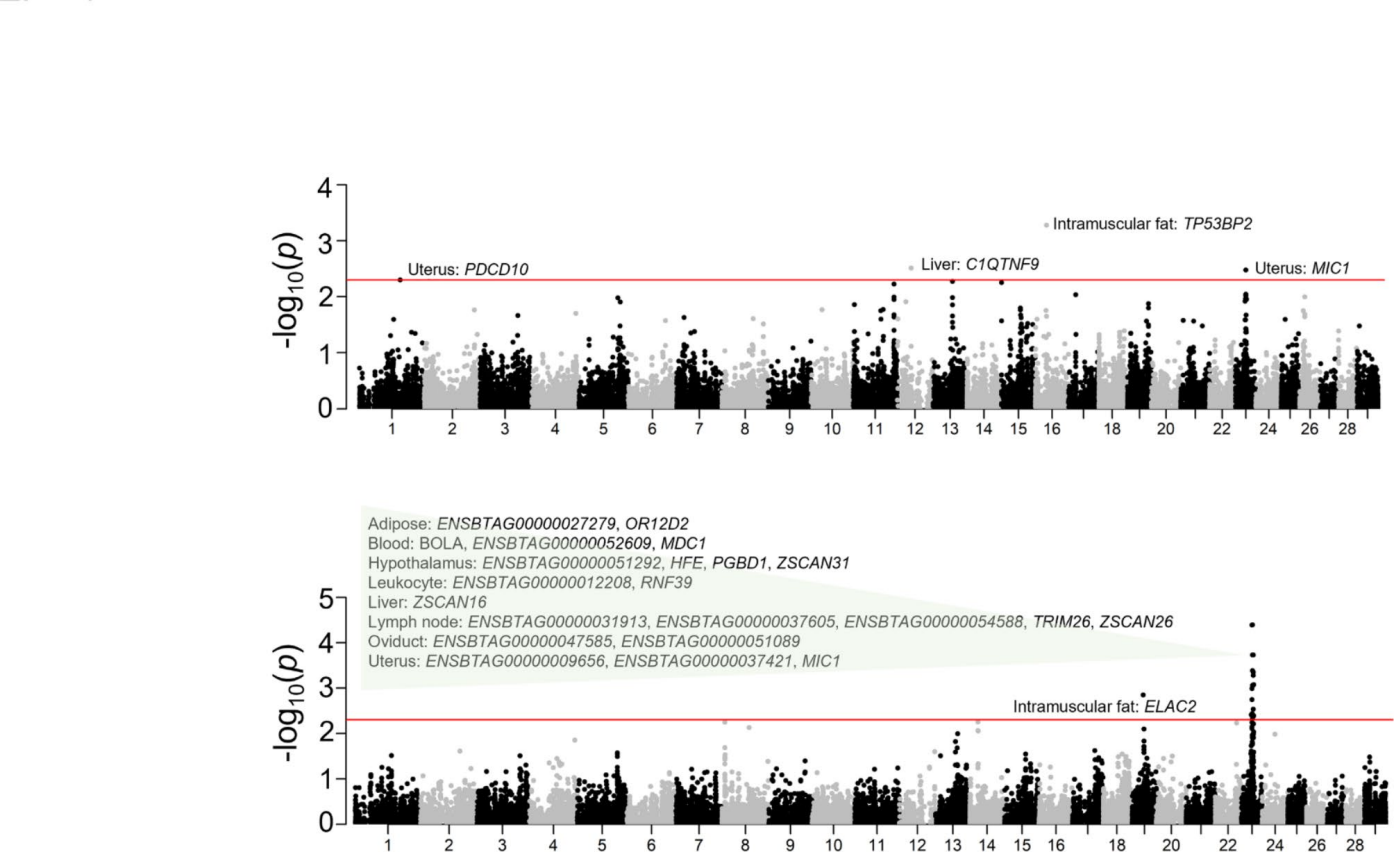
Manhattan plots of transcriptome-wide association study (TWAS) for heifer livability and early calving

TWAS seeks to identify trait-associated genes by testing for the association between a phenotype and the genetic components of gene expression levels [29]. By linking our GWAS results and existing transcriptome data from the CattleGTEx project [30] via a TWAS analysis, we detected four and 23 significant gene-trait association pairs for heifer livability and early calving, respectively (Fig. 4). Interestingly, we discovered six genes overlapped with 27 K bulls GWAS results for early calving in the Bovine MHC region, including two genes in lymph node tissue and one gene in blood, adipose, hypothalamus, and leukocyte (Table 3). In addition, the expression of *OR12D2* in adipose was significantly associated with early calving (Table 3), consistent with previous findings that *OR12D2* is linked with MHC [31].

**Table 3:** Gene-trait association pairs detected by TWAS based on CattleGTEx database

Trait	Gene	Chr	Start	End	*P* value	#SNP	Tissue
Heifer Livability	*TP53BP2*	16	27,139,848	27,207,478	0.000527	18	Intramuscular fat
	*C1QTNF9*	12	34,293,438	34,304,505	0.003071	52	Liver
	*MIC1*	23	27,841,095	27,913,198	0.003337	4	Uterus
	*PDCD10*	1	99,744,376	99,804,996	0.004992	1	Uterus
Early Calving		23	28,925,617	28,926,246	4.00E-05	2	Lymph node
		23	27,796,195	27,797,556	4.09E-05	1	Lymph node
		23	29,612,534	29,613,169	0.000185	1	Adipose
	*ZSCAN26*	23	30,416,140	30,427,406	0.000185	1	Lymph node
	*PGBD1*	23	30,390,348	30,412,787	0.000185	1	Hypothalamus
	*TRIM26*	23	28,777,770	28,787,696	0.000185	1	Lymph node
		23	29,930,410	29,931,333	0.000187	1	Oviduct
	*BOLA*	23	28,720,501	28,724,399	0.000413	5	Blood
	*ZSCAN16*	23	30,561,557	30,568,915	0.000448	1	Liver
	*ZSCAN31*	23	30,377,190	30,379,817	0.000526	2	Hypothalamus
	*H4C3*	23	31,847,243	31,847,554	0.000836	2	Blood
		23	28,677,524	28,686,666	0.000877	1	Uterus
	*OR12D2*	23	29,305,933	29,309,785	0.000878	2	Adipose
	*MIC1*	23	27,841,095	27,913,198	0.001024	4	Uterus
	*ELAC2*	19	31,362,746	31,378,583	0.001419	19	Intramuscular fat
		23	27,871,206	27,875,056	0.001801	1	Leukocyte
		23	30,510,284	30,513,892	0.002936	1	Hypothalamus
	*MDC1*	23	28,304,399	28,316,822	0.003641	3	Blood
		23	25,691,259	25,695,296	0.003865	1	Lymph node
		23	25,583,083	25,589,209	0.003865	1	Uterus
	*HFE*	23	31,855,234	31,864,562	0.004076	1	Hypothalamus
	*RNF39*	23	28,904,289	28,908,861	0.00474	1	Leukocyte
		23	28,741,064	28,750,116	0.00474	1	Oviduct

## Discussion

In this study, we identified genomic regions and candidate genes associated with heifer livability and early first calving using large-scale GWAS, functional enrichment analysis, and TWAS. We reported a major QTL in the bovine MHC region to be related to early first calving, suggesting a potential connection between the immune system and reproduction. TWAS using the CattleGTEx data confirmed the association and revealed several candidate genes in the bovine MHC locus.

Generally, health and fertility traits are expected to be complex traits with low heritability due to their multifactorial nature [32]. The two traits analyzed in this study, heifer livability and early first calving, also have low estimated heritability, 0.0223 and 0.0328, respectively. These two heifer traits can be more complex due to the unique characteristics of heifers. For instance, heifers are not yet fully developed and may not be ready for reproduction. With the large sample sizes used in this study, we only found one major QTL for early first calving but none for heifer livability. Still, when we explored the genome-wide enrichment of GWAS signals with functional genomic regions, we reported significant enrichment of the association signals in promoter and enhancer regions, indicating an exciting connection between the regulation of gene expression and these two complex traits.

The immune system plays a central role in protecting the body from pathogens and infections. Moreover, it has a role in fertility and reproduction. In females, the immune system is involved in the whole reproductive process, from the development and maturation of the egg to implantation and maintenance of the pregnancy [33]. The immune system must tolerate the developing fetus, which is genetically different from the mother, while still protecting against infections. If the immune system is overactive, it can cause infertility or miscarriage, while an underactive immune system can lead to increased susceptibility to infections and complications during pregnancy. These potential connections between the immune system and reproduction further support the MHC QTL and candidate genes with early first calving in heifers.

This study showcased the usefulness of functional genomics data in post-GWAS and fine-mapping studies in cattle. When the associated variants are located in non-coding or intergenic regions, functional genomics data like those from the FAANG [27] and CattleGTEx projects would be useful to provide information about the biological mechanisms underlying the associations. Integration of functional genomics data with GWAS may also boost the power of detection when the power of the original GWAS is limited. For instance, the TWAS results in this research provided additional evidence for the MHC QTL with early first calving.

## Conclusion

Due to the complex genetic architecture of health and fertility traits, our large-scale GWAS analyses only detected a few major QTL for heifer livability and early first calving. Interestingly, the major QTL for early first calving is located in the bovine MHC region. This association was further supported by post-GWAS analyses and TWAS, indicating a connection between the immune system and early reproduction. Despite the low power for major QTL, we evaluated the distribution of GWAS signals across different functional genomic regions. We found significant enrichment in promoter and enhancer-related regions, which supports the contribution of gene regulation to the genetics of complex traits.

## Methods

### Data description

In this study, we conducted GWAS analyses with two datasets, a discovery dataset including 3,649,734 Holstein cattle (336,386 bulls and 3,313,348 cows) genotyped by various SNP chips and imputed to 79,060 SNPs and a fine-mapping dataset including 27,235 bulls genotyped by 50 K SNP chips and imputed to 3,148,506 sequence variants. The original SNP data of the discovery dataset were from multiple SNP chips with densities ranging from 3 to 50 K [34]. The CDCB and USDA AGIL laboratory routinely process the original genotype data and impute to 79 K common SNPs specifically selected for official evaluations using FindHap program [35]. For the discovery dataset, we applied PLINK 1.9 [36] to remove SNPs with call rates < 95%, minor allele frequencies (MAF) < 0.01, Hardy-Weinberg equilibrium (HWE) *P* < 10^− 6^, and to remove animals with > 5% missing genotypes. After this filtering, 73,554 SNPs and 3,520,002 animals (325,905 bulls and 3,194,097 cows) were retained for downstream analyses.

The phenotype data were part of the December 2021 genomic evaluations from the U.S. Council on Dairy Cattle Breeding (CDCB), which routinely calculates predicted transmitting ability (PTA) values for dairy cattle of multiple breeds. We only included Holstein data for this study. We used deregressed PTA values as phenotype in the GWAS of two traits, heifer livability and early first calving [37]. To ensure robustness and accuracy, we excluded animals with low reliability. The majority of filtered animals were young cows without any phenotypic records. Finally, the total number of animals used was 510,318 and 768,645 for heifer livability and early calving, respectively.

For the fine-mapping dataset, we obtained imputed sequence data of 27,235 bulls from previous studies [8]. Briefly, the imputation was conducted with FindHap v3 [35] and 444 Holstein bulls from the Run5 of 1000 Bull Genomes Project as reference. Stringent filtering and removal of intergenic SNPs resulted in an enriched set of 3,148,506 sequence variants. The imputation was highly accurate with an average percentage of consistent genotypes 96.7%. Similarly, we excluded animals with low reliability for deregressed PTA values, retaining 11,562 and 10,700 bulls for heifer livability and early calving, respectively. In this study, we only considered autosomal chromosomes BTA 1–29 from the *Bos taurus* ARS-UCD1.2 assembly [38].

### GWAS analysis

We analyzed the discovery and fine-mapping datasets separately in the GWAS analysis. We performed the GWAS using a linear mixed model approach implemented in the SLEMM program [20]. SLEMM can handle large-scale (up to millions) genome-wide association studies while accounting for genomic relationships. In addition, SLEMM can model differences in the reliability between individual phenotypes using an error weight parameter to account for the variation of deregressed PTAs, which is calculated by 1/*r*^2^-1, where *r*^2^ is the reliability of deregressed PTAs.

After GWAS analysis, we retrieved genes within 1 Mb of the significant SNPs using BioMart in the Ensembl database (Ensembl Genes 106). We carried out Gene Ontology (GO) and Pathway analysis using KOBAS [24]. GO terms with a False Discovery Rate (FDR) less than 5% were considered statistically significant. Furthermore, we compared the regions within 1 Mb of the significant SNPs with our previous GWAS results [8] and the cattle QTLs in the Animal QTL database [25] to check if any associated genomic regions were previously reported.

### Functional enrichment analysis with genome annotations

To evaluate the potential functions of the associated genomic regions, we explored the enrichment of GWAS results in different functional regions using the 27,235 bulls and imputed sequence variants. We performed enrichment analyses via MPH (MINQUE for Partitioning Heritability, https://github.com/jiang18/mph) with the annotations inferred by SnpEff [28] and 14 chromatin states from eight tissues reported by Kern et al. [27]. MPH is designed to partition SNP heritability with genotypes of related individuals or with long-spanning LDs. MPH is comparable to GREML in terms of accuracy, while being much faster and more memory efficient. It can do weighted analyses if residual variances are unequal and use many overlapping functional annotations. This approach included two steps: building genomic relationship matrices (GRMs) based on the different SNP annotation datasets, and partitioning SNP heritability accordingly. We set --min_maf and --min_hwe_pval as 0 and 1e-8 respectively. We calculated standard errors using the Delta method.

### Transcriptome-wide association study (TWAS)

We performed TWAS analyses based on the 27 K bull data using S-PrediXcan [39] to link GWAS results with transcriptome data that we assembled in a previous study [30]. For the TWAS analyses, we used the CattleGTEx v.1 eQTL models [36]. For each trait, we imputed and harmonized GWAS summary statistics and then performed TWAS across 24 cattle tissues separately. We considered genes with *P* < 0.005 as suggestive significant.

### Electronic supplementary material

Below is the link to the electronic supplementary material.

Supplementary Material 1

## Data Availability

The original genotype and phenotype data are owned by third parties and maintained by the Council on Dairy Cattle Breeding (CDCB). A request to CDCB is necessary for getting data access on research, which may be sent to: João Dürr, CDCB Chief Executive Officer (joao.durr@cdcb.us). All other data have been included in the manuscript and supplementary data.

## References

[1] Moorey SE, Biase FH (2020). Beef heifer fertility: importance of management practices and technological advancements. J Anim Sci Biotechnol.

[2] Wathes D, Pollott G, Johnson K, Richardson H, Cooke J (2014). Heifer fertility and carry over consequences for life time production in dairy and beef cattle. Animal.

[3] Krpálková L, Cabrera V, Kvapilík J, Burdych J, Crump P (2014). Associations between age at first calving, rearing average daily weight gain, herd milk yield and dairy herd production, reproduction, and profitability. J Dairy Sci.

[4] Wathes D, Brickell J, Bourne N, Swali A, Cheng Z. Factors influencing heifer survival and fertility on commercial dairy farms. *animal* 2008, 2(8):1135–1143. 10.1017/S1751731108002322 22443725

[5] Kuhn M, Hutchison J, Wiggans G (2006). Characterization of Holstein heifer fertility in the United States. J Dairy Sci.

[6] Zhang H, Wang Y, Chang Y, Luo H, Brito LF, Dong Y, Shi R, Wang Y, Dong G, Liu L (2019). Mortality-culling rates of dairy calves and replacement heifers and its risk factors in Holstein cattle. Animals.

[7] Freebern E, Santos DJ, Fang L, Jiang J, Parker Gaddis KL, Liu GE, VanRaden PM, Maltecca C, Cole JB, Ma L (2020). GWAS and fine-mapping of livability and six Disease traits in Holstein cattle. BMC Genomics.

[8] Jiang J, Cole JB, Freebern E, Da Y, VanRaden PM, Ma L (2019). Functional annotation and bayesian fine-mapping reveals candidate genes for important agronomic traits in Holstein bulls. Commun Biology.

[9] Nayeri S, Sargolzaei M, Abo-Ismail MK, May N, Miller SP, Schenkel F, Moore SS, Stothard P (2016). Genome-wide association for milk production and female fertility traits in Canadian dairy Holstein cattle. BMC Genet.

[10] Tenghe A, Bouwman A, Berglund B, Strandberg E, de Koning D, Veerkamp R (2016). Genome-wide association study for endocrine fertility traits using single nucleotide polymorphism arrays and sequence variants in dairy cattle. J Dairy Sci.

[11] Johnston D, Mukiibi R, Waters SM, McGee M, Surlis C, McClure JC, McClure MC, Todd CG, Earley B (2020). Genome wide association study of passive immunity and Disease traits in beef-suckler and dairy calves on Irish farms. Sci Rep.

[12] Narayana SG, de Jong E, Schenkel FS, Fonseca PA, Chud TC, Powel D, Wachoski-Dark G, Ronksley PE, Miglior F, Orsel K. Underlying genetic architecture of resistance to mastitis in dairy cattle: a systematic review and gene prioritization analysis of genome-wide association studies. J Dairy Sci 2022. 10.3168/jds.2022-21923 36333139

[13] Neupane M, Hutchison J, Van Tassell C, VanRaden P (2021). Genomic evaluation of dairy heifer livability. J Dairy Sci.

[14] Hutchison J, VanRaden P, Null D, Cole J, Bickhart D (2017). Genomic evaluation of age at first calving. J Dairy Sci.

[15] Gulliksen S, Lie K, Løken T, Østerås O (2009). Calf mortality in Norwegian dairy herds. J Dairy Sci.

[16] Fuerst-Waltl B, Sørensen M (2010). Genetic analysis of calf and heifer losses in Danish holstein. J Dairy Sci.

[17] Weller JI, Gershoni M, Ezra E (2021). Genetic and environmental analysis of female calf survival in the Israel Holstein cattle population. J Dairy Sci.

[18] Vergara O, Elzo M, Cerón-Muñoz M (2009). Genetic parameters and genetic trends for age at first calving and calving interval in an Angus-Blanco Orejinegro-Zebu multibreed cattle population in Colombia. Livest Sci.

[19] Grossi D, Venturini G, Paz C, Bezerra L, Lôbo RB, Oliveira J, Munari D (2009). Genetic associations between age at first calving and heifer body weight and scrotal circumference in Nelore cattle. J Anim Breed Genet.

[20] Cheng J, Maltecca C, VanRaden PM, O’Connell JR, Ma L, Jiang J (2023). SLEMM: million-scale genomic predictions with window-based SNP weighting. Bioinformatics.

[21] Ellis SA, Ballingall KT (1999). Cattle MHC: evolution in action?. Immunol Rev.

[22] Nishimura S, Watanabe T, Mizoshita K, Tatsuda K, Fujita T, Watanabe N, Sugimoto Y, Takasuga A (2012). Genome-wide association study identified three major QTL for carcass weight including the PLAG1-CHCHD7 QTN for stature in Japanese black cattle. BMC Genet.

[23] Xu Y, Harder KW, Huntington ND, Hibbs ML, Tarlinton DM (2005). Lyn tyrosine kinase: accentuating the positive and the negative. Immunity.

[24] Xie C, Mao X, Huang J, Ding Y, Wu J, Dong S, Kong L, Gao G, Li C-Y, Wei L (2011). KOBAS 2.0: a web server for annotation and identification of enriched pathways and Diseases. Nucleic Acids Res.

[25] Hu Z-L, Park CA, Reecy JM (2022). Bringing the animal QTLdb and CorrDB into the future: meeting new challenges and providing updated services. Nucleic Acids Res.

[26] Abrams ET, Miller EM (2011). The roles of the immune system in women’s reproduction: evolutionary constraints and life history trade-offs. Am J Phys Anthropol.

[27] Kern C, Wang Y, Xu X, Pan Z, Halstead M, Chanthavixay G, Saelao P, Waters S, Xiang R, Chamberlain A (2021). Functional annotations of three domestic animal genomes provide vital resources for comparative and agricultural research. Nat Commun.

[28] Cingolani P, Platts A, Wang LL, Coon M, Nguyen T, Wang L, Land SJ, Lu X, Ruden DM (2012). A program for annotating and predicting the effects of single nucleotide polymorphisms, SnpEff: SNPs in the genome of Drosophila melanogaster strain w1118; iso-2; iso-3. Fly.

[29] Gusev A, Ko A, Shi H, Bhatia G, Chung W, Penninx BW, Jansen R, De Geus EJ, Boomsma DI, Wright FA (2016). Integrative approaches for large-scale transcriptome-wide association studies. Nat Genet.

[30] Liu S, Gao Y, Canela-Xandri O, Wang S, Yu Y, Cai W, Li B, Xiang R, Chamberlain AJ, Pairo-Castineira E (2022). A multi-tissue atlas of regulatory variants in cattle. Nat Genet.

[31] Younger RM, Amadou C, Bethel G, Ehlers A, Lindahl KF, Forbes S, Horton R, Milne S, Mungall AJ, Trowsdale J (2001). Characterization of clustered MHC-linked olfactory receptor genes in human and mouse. Genome Res.

[32] Ma L, Cole J, Da Y, VanRaden P. Symposium review: Genetics, genome-wide association study, and genetic improvement of dairy fertility traits. *Journal of dairy science* 2019, 102(4):3735–3743. 10.3168/jds.2018-15269 30268602

[33] Lee SK, Kim CJ, Kim D-J, Kang J-h (2015). Immune cells in the female reproductive tract. Immune Netw.

[34] Wiggans G, Cooper T, VanRaden P, Van Tassell C, Bickhart D, Sonstegard T (2016). Increasing the number of single nucleotide polymorphisms used in genomic evaluation of dairy cattle. J Dairy Sci.

[35] VanRaden PM, Sun C, O’Connell JR (2015). Fast imputation using medium or low-coverage sequence data. BMC Genet.

[36] Chang CC, Chow CC, Tellier LC, Vattikuti S, Purcell SM, Lee JJ. Second-generation PLINK: rising to the challenge of larger and richer datasets. *Gigascience* 2015, 4(1):s13742-13015-10047-13748. 10.1186/s13742-015-0047-8 PMC4342193 25722852

[37] Garrick DJ, Taylor JF, Fernando RL (2009). Deregressing estimated breeding values and weighting information for genomic regression analyses. Genet Selection Evol.

[38] Rosen BD, Bickhart DM, Schnabel RD, Koren S, Elsik CG, Tseng E, Rowan TN, Low WY, Zimin A, Couldrey C (2020). De novo assembly of the cattle reference genome with single-molecule sequencing. Gigascience.

[39] Barbeira AN, Dickinson SP, Bonazzola R, Zheng J, Wheeler HE, Torres JM, Torstenson ES, Shah KP, Garcia T, Edwards TL (2018). Exploring the phenotypic consequences of tissue specific gene expression variation inferred from GWAS summary statistics. Nat Commun.

